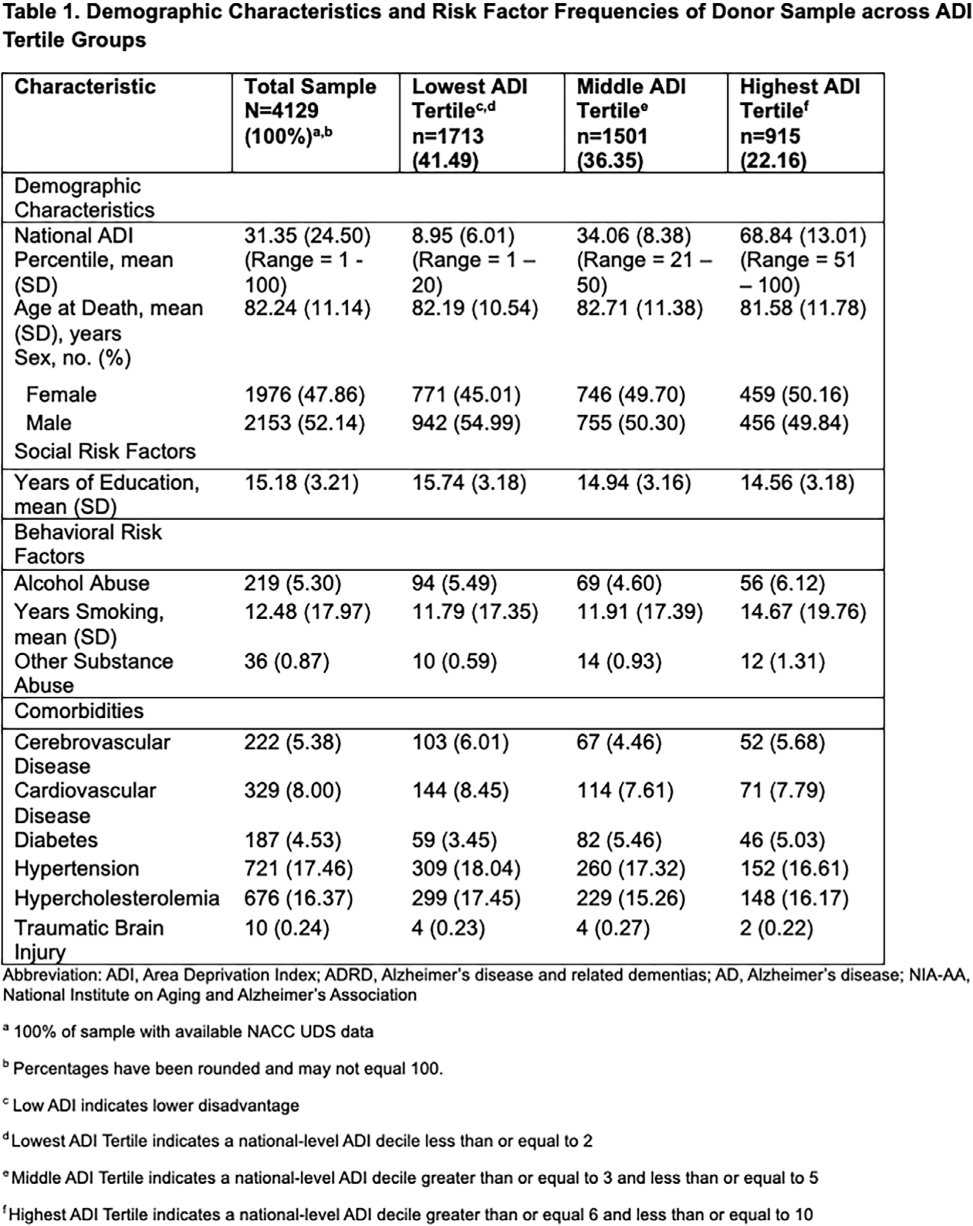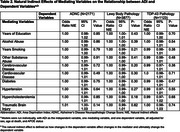# Challenges of mediation analysis in a national sample of brain bank donors

**DOI:** 10.1002/alz70860_103598

**Published:** 2025-12-23

**Authors:** Sarah A. Keller, W. Ryan Powell, Grace C George, William R. Buckingham, Amy J.H. Kind, the Neighborhoods Study Investigator Team

**Affiliations:** ^1^ Center for Health Disparities Research, University of Wisconsin School of Medicine and Public Health, Madison, WI, USA; ^2^ Department of Medicine, Geriatrics Division, School of Medicine and Public Health, University of Wisconsin‐Madison, Madison, WI, USA; ^3^ Wisconsin Alzheimer's Disease Research Center, School of Medicine and Public Health, University of Wisconsin‐Madison, Madison, WI, USA

## Abstract

**Background:**

Previous work has established relationships between the adverse social exposome, measured by the Area Deprivation Index (ADI), and Alzheimer's disease (AD), TDP‐43, and Lewy body neuropathology, but the mediating effects along the causal pathways underlying these associations are unknown. Mediation analysis clarifies how an exogenous or independent variable (IV) affects a dependent variable (DV) through an endogenous variable known as a mediator. Objective: test for effect modification between ADI and AD, TDP‐43, and Lewy body neuropathology in a national brain bank sample from the multisite Neighborhoods Study.

**Method:**

All brain donors from participating sites with National Alzheimer's Coordinating Center (NACC) neuropathology data and geocodable addresses (*N* = 8637) were eligible. The presence of social, behavioral, and comorbid conditions as AD risk factors were drawn from the NACC uniform dataset (UDS) (*n* = 4129). Addresses were linked to ADIs corresponding to year of death and grouped into tertiles. Causal mediation analysis was performed, with ADI as the IV; AD, TDP‐43, and Lewy body neuropathology defined as binary DVs; and AD social and behavioral risk factors and comorbid conditions as mediators in individual models, adjusted for sex, age at death, and APOE status.

**Result:**

Availability of neuropathologic assessments varied across the DVs of interest: AD (*n* = 2171), TDP‐43 (*n* = 1123), and Lewy body (*n* = 3877) (Table 1). None of the AD risk factors were shown to have a significant mediating effect on the association between ADI and the Neuropathology (Table 2). Post‐hoc Monte Carlo analyses, which allows researchers to tailor power analyses to their models by specifying all parameter values, found that samples were underpowered to detect effects.

**Conclusion:**

Several possibilities exist for failing to find a mediating effect. Additional efforts to mitigate unmeasured confounders, one of the major assumptions of causal mediation analysis, is possible. Identifying additional mediators on the causal pathway, such as exposure to heavy metals, or increasing UDS assessments for AD risk factors could improve power, could be beneficial. More work is necessary to identify all variables on the causal pathway and improve statistical power to understand the relationship between the social exposome and neuropathology.